# The United States of America and Scientific Research

**DOI:** 10.1371/journal.pone.0012203

**Published:** 2010-08-16

**Authors:** Gregory J. Hather, Winston Haynes, Roger Higdon, Natali Kolker, Elizabeth A. Stewart, Peter Arzberger, Patrick Chain, Dawn Field, B. Robert Franza, Biaoyang Lin, Folker Meyer, Vural Ozdemir, Charles V. Smith, Gerald van Belle, John Wooley, Eugene Kolker

**Affiliations:** 1 Bioinformatics & High-throughput Analysis Laboratory, Seattle Children's Research Institute, Seattle, Washington, United States of America; 2 High-throughput Analysis Core, Seattle Children's Research Institute, Seattle, Washington, United States of America; 3 Hendrix College, Conway, Arkansas, United States of America; 4 Predictive Analytics, Seattle Children's Hospital, Seattle, Washington, United States of America; 5 Center for Research on BioSystems, University of California San Diego, San Diego, California, United States of America; 6 Bioscience Division, Los Alamos National Laboratory, Los Alamos, New Mexico, United States of America; 7 Metagenomics Program, Joint Genome Institute, Walnut Creek, California, United States of America; 8 Center for Microbial Ecology, Michigan State University, East Lansing, Michigan, United States of America; 9 Centre for Ecology and Hydrology, Natural Environmental Research Council, Oxford, United Kingdom; 10 MYOONET, Inc., Seattle, Washington, United States of America; 11 Department of Bioengineering, University of Washington, Seattle, Washington, United States of America; 12 Zhejiang-California International Nanosystems Institute, Zhejiang University, Hangzhou, China; 13 Swedish Neuroscience Institute, Swedish Medical Center, Seattle, Washington, United States of America; 14 Department of Urology, University of Washington, Seattle, Washington, United States of America; 15 Argonne National Laboratory, Argonne, Illinois, United States of America; 16 University of Chicago, Chicago, Illinois, United States of America; 17 Department of Human Genetics, Faculty of Medicine, McGill University, Montreal, Quebec, Canada; 18 Center for Developmental Therapeutics, Seattle Children's Research Institute, Seattle, Washington, United States of America; 19 Department of Pediatrics, University of Washington, Seattle, Washington, United States of America; 20 Department of Biostatistics, University of Washington, Seattle, Washington, United States of America; 21 Department of Environmental and Occupational Health Sciences, University of Washington, Seattle, Washington, United States of America; 22 Department of Medical Education and Biomedical Informatics, University of Washington, Seattle, Washington, United States of America; Cuban Neuroscience Center, Cuba

## Abstract

To gauge the current commitment to scientific research in the United States of America (US), we compared federal research funding (FRF) with the US gross domestic product (GDP) and industry research spending during the past six decades. In order to address the recent globalization of scientific research, we also focused on four key indicators of research activities: research and development (R&D) funding, total science and engineering doctoral degrees, patents, and scientific publications. We compared these indicators across three major population and economic regions: the US, the European Union (EU) and the People's Republic of China (China) over the past decade. We discovered a number of interesting trends with direct relevance for science policy. The level of US FRF has varied between 0.2% and 0.6% of the GDP during the last six decades. Since the 1960s, the US FRF contribution has fallen from twice that of industrial research funding to roughly equal. Also, in the last two decades, the portion of the US government R&D spending devoted to research has increased. Although well below the US and the EU in overall funding, the current growth rate for R&D funding in China greatly exceeds that of both. Finally, the EU currently produces more science and engineering doctoral graduates and scientific publications than the US in absolute terms, but not per capita. This study's aim is to facilitate a serious discussion of key questions by the research community and federal policy makers. In particular, our results raise two questions with respect to: a) the increasing globalization of science: “What role is the US playing now, and what role will it play in the future of international science?”; and b) the ability to produce beneficial innovations for society: “How will the US continue to foster its strengths?”

## Introduction

Research in the US is widely believed to be essential to the country's economic growth, and the innovations derived from basic and applied research provide enormous benefits to society [1]. For this reason, the federal government devotes a significant amount of funding towards research [1]–[10]. A number of economic studies are aimed at quantifying the benefit of federal research spending in terms of the return on investment [2], [11]–[15] or the contribution to the growth of the economy [16]. These studies, among others, include some detailed estimates of the optimal amount of spending on research [16], [17]. A large and ever growing number of projects developed with FRF have provided great benefits to society. Two notable and widely cited examples of applied and basic research, respectively, are the laser [18] and the PageRank algorithm [19]. The laser, developed with Federal support since the 1950s, was initially seen only as an academic “solution waiting for a problem” [18], but now it has a growing number of applications (for example, telecommunications and medical technologies) benefiting diverse aspects of our lives. More recently, the PageRank algorithm [19] was developed with support from the National Science Foundation (NSF) and the Department of Defense's DARPA (Defense Advanced Research Projects Agency, www.darpa.mil) and, eventually, led to the formation of Google, the multi-billion dollar internet corporation, whose products are used throughout the world.

In this study, we seek to provide an objective analysis of the state of scientific research: a) in the US since the 1960s and b) in comparison with two other major population and economic regions, the EU (specifically, the EU-27, which consists of Austria, Belgium, Bulgaria, Cyprus, the Czech Republic, Denmark, Estonia, Finland, France, Germany, Greece, Hungary, Ireland, Italy, Latvia, Lithuania, Luxembourg, Malta, the Netherlands, Portugal, Romania, Slovak Republic, Slovenia, Spain, Sweden, and the United Kingdom) and China since 1996. This study focuses only on the comparison between these three population (over 300 million people each) and economic (over half of the US GDP each) regions since they are the only regions with both this level of population and economic output.

The task of evaluating scientific output using quantitative measures has been widely discussed [20]–[34]. The four indicators selected for our comparison are: 1. federal R&D spending; 2. the number of science and engineering doctorates awarded; 3. the number of patents issued; and 4. the number of papers published. The four metrics we chose for consideration have been utilized and evaluated in multiple studies [20]–[25]. These studies have also brought into discussion other important indicators such as citations, number of researchers, exports, and international prizes. While many of these metrics may have validity, we have attempted to simplify the comparisons by choosing the four most robust indicators having the most reliable and consistently available datasets. Our analysis relies purely on quantitative data because, though attempts have been made to develop a qualitative analysis, the low availability and inherent subjectivity of qualitative data make such an analysis exceptionally difficult [24]. Future analyses should address the more difficult problem of measuring research quality. However, our analysis is aimed at combining these four metrics in a unique manner which develops an understanding of the US's current and future role in the scientific research community, a perspective which has not yet been discussed in the literature.

Work involving scientific and technical innovation is often divided into basic research, applied research and development. According to the NSF, basic research is the study of phenomena without specific applications in mind, while applied research is study to gain knowledge necessary to determine how a specific need may be met [3]. Development is the application of knowledge to produce useful devices to meet specific requirements [3]. In the present work, we do not distinguish between different types of research, and focus on research overall *vs.* R&D.

The relative research statuses of China, the EU, and the US in the world's scientific research community are popular topics in literature. China's increasing output is undeniable and its impact on the global landscape is widely discussed [25]–[33]. One fierce debate is between experts who believe China poses a serious threat to American scientific prowess and others who are more dismissive of the threat of Chinese scientific advance. The relative importance of European science is another contentious area in which scholars add to the debate ideas about the relative importance of different research fields [23], [24]. The spread of globalization and increase in international collaborations has several researchers worried that the US is not receiving full credit for its research contributions [28], [34]. Concern about US competitiveness in this changing research climate has been discussed in a number of notable reports issued by the Congressional Budget Office [2], [4], the National Academies [5], the Battelle Memorial Institute [6], and the NSF [3], [7]–[10]. Several authors evaluated trends in federal research spending [35], leading some of them to call for increases in either US federal or private R&D funding [21], [36]. Many other nations have instituted policies designed to encourage innovation in all sectors [37]. Some analysts view the relative increase of scientific productivity in other countries as a threat to the US, but our view is that the exchange of scientific knowledge has the potential to benefit all countries involved [38]. From this wealth of opinions and fears about a US decline in scientific prowess we have attempted to draw rational conclusions to guide further discussion among scientists and policymakers. This study examines data from relevant sources and describes key research indicators for the US (over the past six decades), and the EU-27 and China (over the last decade).

## Methods

### Data and Analysis

#### Research spending

Table 1 lists the datasets analyzed in this paper from numerous reliable sources, including several NSF surveys. The first [3] was a survey of all federal agencies that funded basic and applied research. The second [8] was a sample survey of organizations that perform research, and it requested the outlays for basic and applied research, and for the origin(s) of the funding to be specified. For example, an institution could spend money on a research project, but funding for the project could come from a contract with the federal government [8]. The companies included in this survey were categorized by the type of industry to which they belonged [10].

**Table 1:** Datasets used in the analysis.

Dataset	Source organization and reference	Figures	Table
Federal funds for R & D	NSF [3]	1, 2, 3	3
Gross domestic product	Bureau of Economic Analysis [52]	1, 3	
Consumer price index	Bureau of Labor Statistics [53]	1, 2, 3	
Budget of the US government	OMB [39]	1, 3, 4	
National patterns of R&D resources	NSF [8]	5, 6	
Survey of industrial R&D	NSF [10]		2
Science and technology tables	OECD [40]–[43]	7, 8	
Graduates by field of education	OECD [46]	9, 10	
Science and engineering indicators	NSF [7]	9, 10	
Triadic patents granted	OECD [45]	11, 12	
US population	US Census Bureau [54], [55]	2, 8, 10, 12, 14	
EU-15 population	European Central Bank [56]	8, 10, 12, 14	

From the Office of Management and Budget (OMB) [39], we obtained a historical record of the US federal budget that contains the total outlays since 1956 (see Table 1). In addition, since 1962, OMB recorded mandatory and discretionary outlays for each federal program. Discretionary spending is set by the US government each fiscal year and includes, for example, defense, education and research spending [39]. In contrast, mandatory spending is not set by an annual spending bill; it includes Social Security and Medicare.

We used multiple datasets made available from the Organization for Economic Co-operation and Development (OECD) from 1996–2006 for China, the EU-27, and the US. These datasets included: R&D spending, R&D spending divided by GDP, R&D spending divided by population, and percent of R&D financed by government [40]–[43].

#### Patents

To rule out the potential bias in domestic patents discussed at length in [44], we utilized triadic patent data. Triadic patents are patents which are valid with the United States Patent and Trademark Office, the Japan Patent Office, and the European Patent Office. These patents will theoretically represent higher value discoveries. Though these patents are not valid in China and many other countries, they present the closest reality to an international patent. The triadic patent data from 1996–2006 was accessed through OECD for China, the EU-27, and the US [45].

#### Doctoral degrees

For our analysis of doctoral degrees, we found the number of science and engineering doctorates awarded by institutions in China, the EU, and the US [7]. (Due to data constraints, analysis for doctoral degrees was limited to the EU-15 rather than the EU-27.) We obtained the number of science and engineering doctorates awarded by the EU countries from the OECD [46]. To standardize these datasets as much as possible (Table 1), we selected the degrees classified with the UNESCO International Standard Classification of Education 1997 [47] (field codes 31, 42, 44, 46, 48, 52, 54, 58, and 62) as representing science and engineering degrees. (Missing values were set to 0.)

#### Papers

We also analyzed the number of papers produced by authors in the US, the EU-27 and China [7] from journals covered in the *Thomson Reuters* Science Citation Index or the Social Sciences Citation Index. Each region received a fractional count based on the fraction of the institutions in the region.

#### Limitations of the data and analysis

Datasets have been acquired from the best and most reliable sources available. However, there are some issues with regard to comparability across regions. In particular, the data do not measure the skill level of the doctoral graduates, the value of the patents granted or the originality of the papers published. These qualities may vary between the regions. Despite these potential limitations, the comparisons are illustrative in the present context.

Most of the datasets were directly amenable to statistical analysis, including, for example, the annual absolute and relative differentials. The OECD data had missing values for the fraction of R&D funded by the government in China for 2001 and 2002 therefore we estimated this using linear interpolation.

## Results

### State of the research support in the US

**Figure 1:**
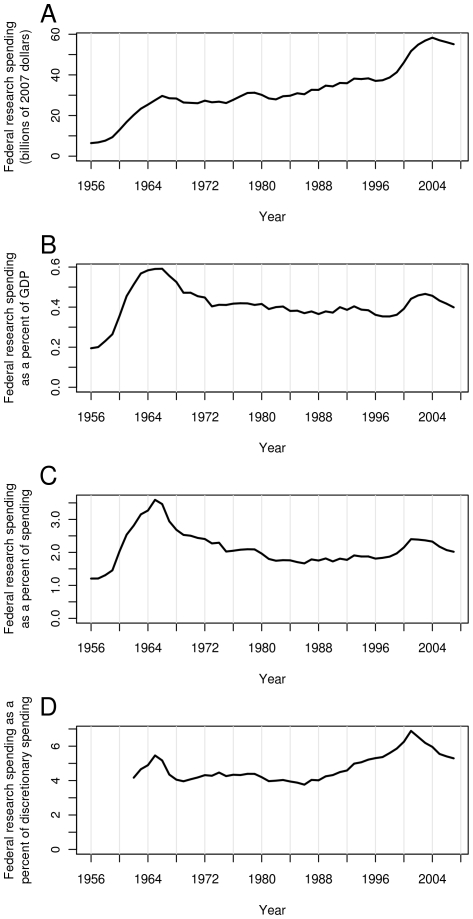
Federal research funding (FRF) in the United States of America (US) during the last six decades. FRF in constant dollars (A), as a percent of gross domestic product (GDP) (B), as a percent of federal spending (C), and as a percent of federal discretionary spending (D). Discretionary spending is money which is set aside each fiscal year by an annual spending bill on a non-mandatory basis.

**Figure 2:**
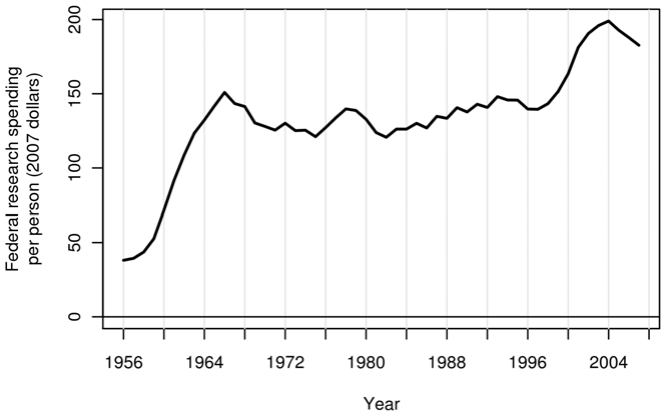
Per capita FRF in the US during the last six decades. FRF in inflation adjusted dollars per person.

**Figure 3:**
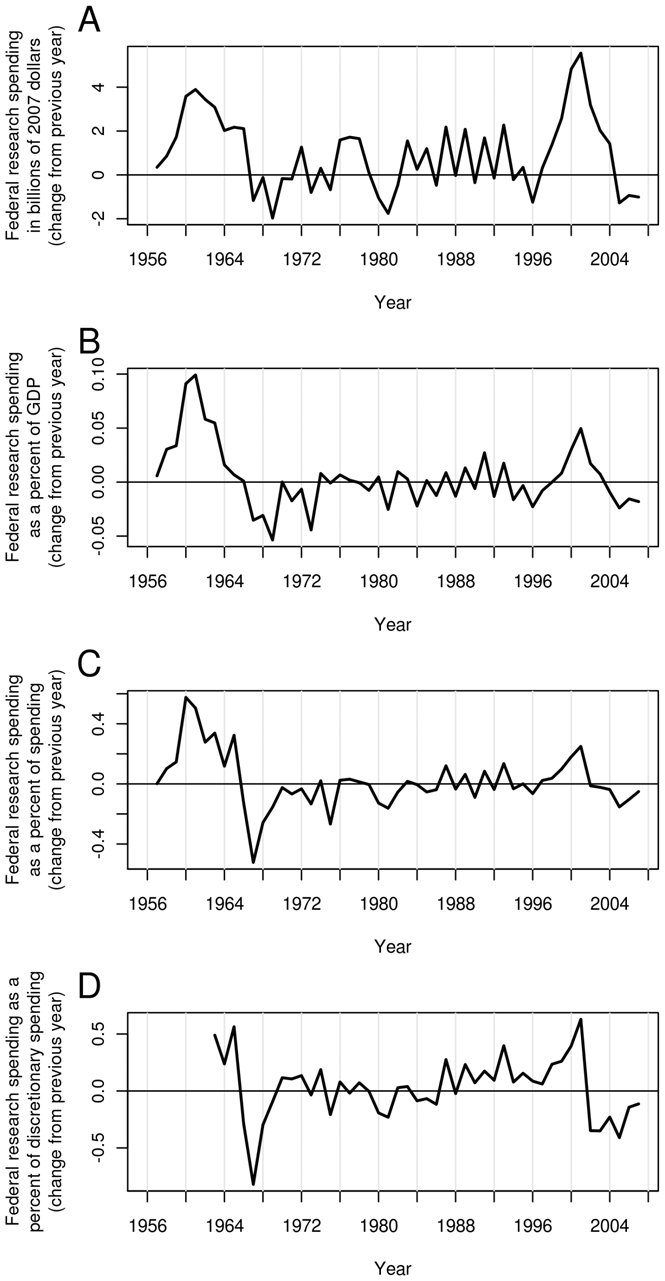
Change in FRF in the US during the last six decades. Each point on the graph represents the difference in FRF between consecutive years. The change in FRF can be viewed in inflation adjusted dollars (A), as a percent of GDP (B), as a percent of federal spending (C), and as a percent of federal discretionary spending (D).

Adjusted for inflation and divided by the population, growth in the US FRF has been uneven, with large increases during some years and gradual reductions during other years (Figures 1A and 2). Figure 1 also shows FRF as a percentage of GDP (B), total federal spending (C) and federal discretionary spending (D). Each plot shows two peaks, one during the mid 1960s, and the other during the early 2000s. Year to year differences for the above quantities are shown in Figure 3.

**Figure 4:**
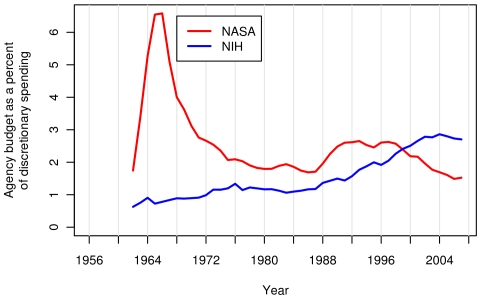
National Aeronautics and Space Administration (NASA) and National Institutes of Health (NIH) budgets during the last six decades. Budgets for NASA and NIH as a percent of US federal discretionary spending.

The two peaks can be explained mainly by the trends in the National Aeronautics and Space Administration (NASA) and National Institutes of Health (NIH) budgets. The first peak is attributable to the burst of funding that NASA received during the space race, and the second peak to the period of regularly increased NIH funding on the “doubling curve” (Figure 4). Which of these peaks is higher depends on whether one considers total or discretionary spending. The curves have different shapes in part because discretionary spending has declined from two-thirds of the federal budget in 1965 to one-third in 2001. Therefore, the FRF surges had different impacts on the two budgets. In either case, the fraction of spending devoted to research has declined since 2001.

**Figure 5:**
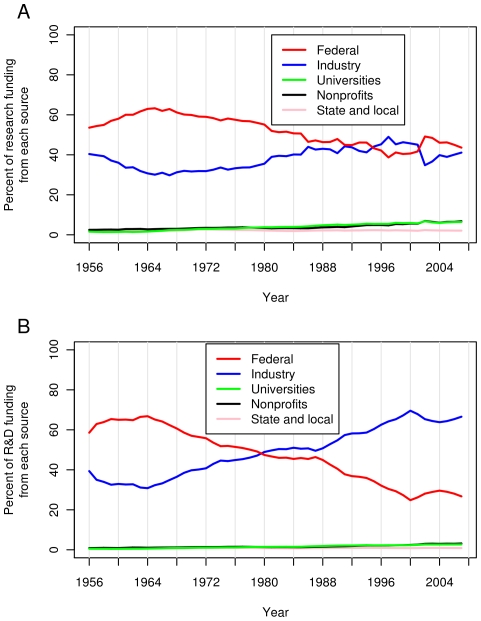
Five sources of research and research and development (R&D) funding in the US during the last six decades. Percentage of research (A) and R&D (B) spending derived from five different sources: 1. federal agencies, 2. industry, 3. universities and colleges, 4. nonprofit organizations, and 5. state and local governments.

**Figure 6:**
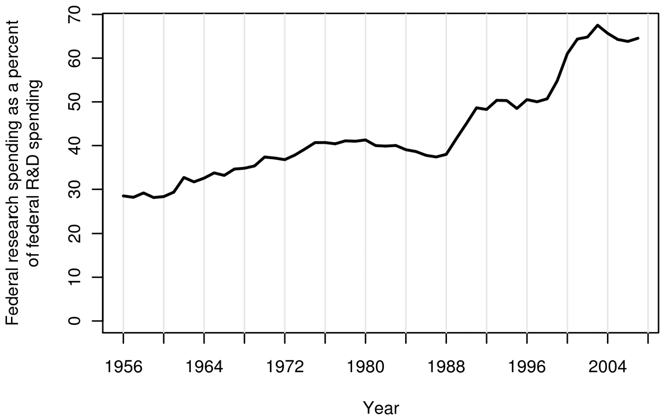
The US FRF as a percent of federal R&D spending. The research portion of federal R&D spending in the US.

Figure 5A compares US FRF with research funding from other sources since 1956. At present, industry and the federal government contribute approximately equally towards research (the FRF is 106% that of industry). However, 45 years ago, FRF was more than twice (221%) that of industry. Although contributions from nonprofits, universities and state governments have increased slowly, these contributions still account for less than 16% of total research spending in the US. As shown in Figure 5B, when R&D spending is considered (i.e., development spending is added), industry's spending has grown from half (49.6% in 1965) to more than two times (249% in 2007) that of the government. This change is in part the result of a shift from development spending to research spending by the US government (Figure 6). US FRF has risen from 33.7% in 1965 of the total US R&D spending to 64.6% in 2007 (Figure 6).

Table 2 summarizes the distribution of funds spent by industry on R&D in 2007, while Table 3 does the same for the federal government R&D. It is interesting to note that among manufacturing industries, which capture 69.8% of the overall industry spending on R&D, pharmaceuticals and medicines account for 19.6% of the overall spending. This reflects both the importance of Healthcare to the overall US economy and the research intensive nature of this industry (only the category of Computers and electronic parts ranked higher in spending). Among nonmanufacturing industries (36.2% of the spending), scientific R&D services capture only 5% of the overall R&D spending (Table 2).

**Table 2:** Breakdown of industry spending on R&D in 2007.

Category	Subcategory	Further subcategory	Percent
Manufacturing			69.8
	Chemicals		22.8
		Pharmaceuticals and medicines	19.6
	Computer and electronic products		20.5
		Semiconductor and other electronic components	7.5
		Navigational, measuring, electromedical, and control instruments	5.1
	Transportation equipment		12.8
		Motor vehicles, trailers, and parts	6.6
		Aerospace products and parts	5.5
Nonmanufacturing			30.2
	Information		11.9
		Publishing, including software	8.6
	Professional, scientific, and technical services		13.6
		Computer systems design and related services	5.6
		Scientific R&D services	5.0

Only categories and subcategories that received at least 5% of the overall industry R&D spending are listed.

**Table 3:** Breakdown of federal spending on R&D in 2007.

Agency	Subordinate agency	Percent
Department of Defense		49.9
	DARPA	3.1
	Missile Defense Agency	8.0
	Department of the Air Force	10.3
	Department of the Army	9.3
	Department of the Navy	15.3
Department of Education		0.3
Department of Energy		7.1
Department of Health and Human Services		25.6
	National Institutes of Health	24.5
National Aeronautics and Space Administration		7.2
National Science Foundation		3.6

Agencies that received at least 5% of the overall federal R&D spending are listed. In addition, we included the Defense Advanced Research Projects Agency (DARPA), the Department of Education and the National Science Foundation (NSF).

In contrast, 75.5% of federal R&D spending is captured by two categories: the Department of Defense (49.9%) and the NIH (24.5%) within the Department of Health and Human Services (Table 3). Surprisingly, DARPA receives only 3.1% of the federal R&D budget yet has generated a great number of high impact science and technology advances. Also surprisingly, the NSF, a pivotal funding agency for the scientific community charged with promoting the progress of basic and applied science, receives only 3.6%. Finally, the Department of Education, which is responsible for the advancement of teaching methods for the future workforce, receives only 0.3% of the federal R&D budget.

### Research indicators in the US, the EU and China

**Figure 7:**
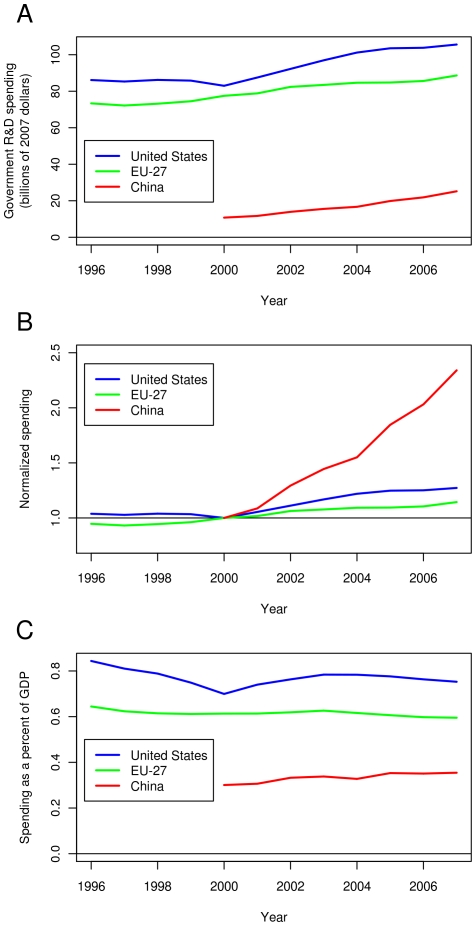
Recent government spending on research and development in the US, the European Union (EU-27) and People's Republic of China (China). Government R&D spending adjusted for inflation (A). Government R&D spending normalized by adjusting for inflation and scaling to have unit spent equivalent to that country's R&D spending in 2000 (B). Government R&D as a percent of that nation's GDP (C).

**Figure 8:**
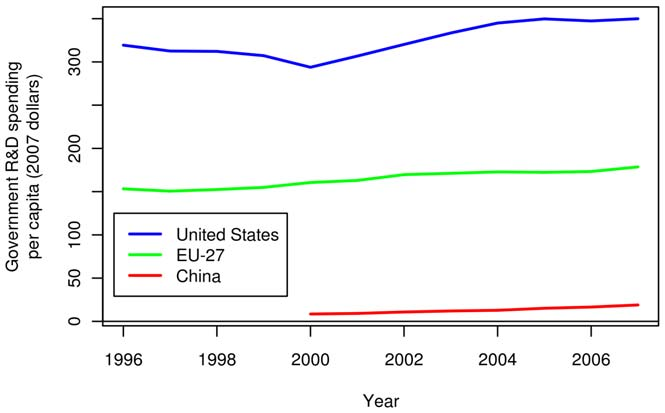
Per capita government spending on research and development in the US, the EU-27 and China. Federal government spending on R&D per person, in inflation adjusted dollars, from 1996 to 2007 for the US, the EU-27 and China. Per capita figures were divided by the population of the region.

Figure 7A shows absolute government R&D spending in the US, the EU-27 and China from 1996 to 2007. Currently, the US still has a higher level of R&D spending than the EU-27 and China. The same holds at a per capita level, as shown in Figure 8. Although R&D spending is much lower in China than in the US and the EU-27, their growth rate of spending relative to 2000 is much higher than the US or the EU-27 (Figure 7B). Figure 7C shows government R&D spending as a percent of GDP. Both the US and the EU-27 R&D budgets as fractions of their respective GDPs have been declining in recent years, while China's has held steady; however, China's recent GDP growth has been much greater than that of the US or the EU-27. Therefore, in absolute numbers, a constant fraction of the Chinese GDP is a substantial increase, in absolute terms, for China's FRF.

**Figure 9:**
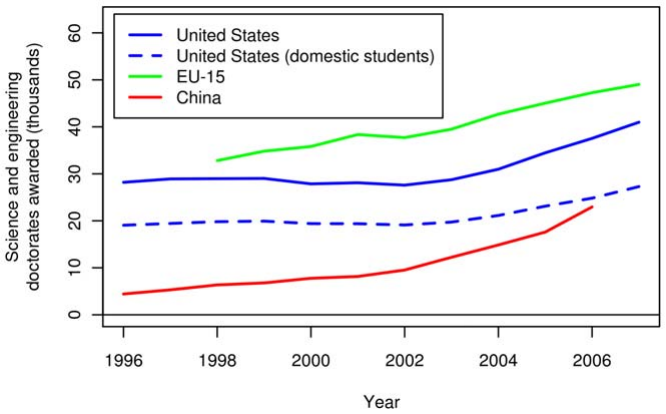
Recent science and engineering doctoral awards in the US, the EU-15 and China. The solid lines show the number of doctoral degrees granted by institutions in each region. In addition, the dashed line shows the number of doctoral degrees granted by the US institutions to the US citizens and permanent residents.

**Figure 10:**
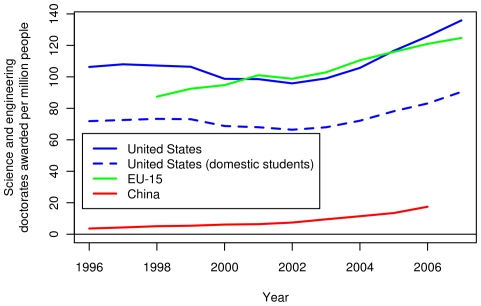
Per capita science and engineering doctoral awards in the US, the EU-15 and China. The solid lines show the number of doctoral degrees granted per capita by institutions in the US, the EU-15 and China. In addition, the dashed line shows the number of doctoral degrees granted per capita by the US institutions to the US citizens and permanent residents. Per capita figures were divided by the population of the region.

Figure 9 shows the number of science and engineering doctorates awarded in the US, the EU and China. Figure 10 displays these quantities per capita. Over the range for which data are available, the EU-27 produced the most science and engineering doctorates in absolute terms, followed by the US and China. However, the US still produces the most science and engineering doctorates per capita. Figure 9 also shows the number of science and engineering doctoral degrees awarded in the US to its citizens and permanent residents. All of the curves, except the one for China, show a dip in 2002 and steady growth thereafter.

We make three key observations from Figures 9 and 10. First, the EU-15 produces the highest number of science and engineering doctorate degrees. Second, although the number of graduates in China started at a very low level, it increased by a factor of five from 1996 to 2006. This number does not include the number of Chinese who obtain their doctorates overseas and return home (this number was not available). However, an estimated 4,500 Chinese students obtained science and engineering doctoral degrees from the US in 2007 [7]. Third, the number of US degrees awarded to US citizens and permanent residents (solid and dashed lines, respectively) has consistently been about two-thirds of the total number of degrees awarded in the US. The rest are awarded to students with temporary visas. A recent study [48] estimated that 40% of these students return home within five years of graduation.

**Figure 11:**
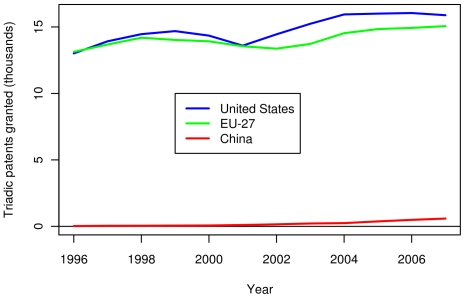
Recent triadic patents granted in the US, the EU-27 and China. Triadic patents are patents which are valid with the United States Patent and Trademark Office, the Japan Patent Office, and the European Patent Office. The blue, green, and red lines show the number of triadic patents granted to American (US), European (within the EU-27), and Chinese inventors, respectively.

**Figure 12:**
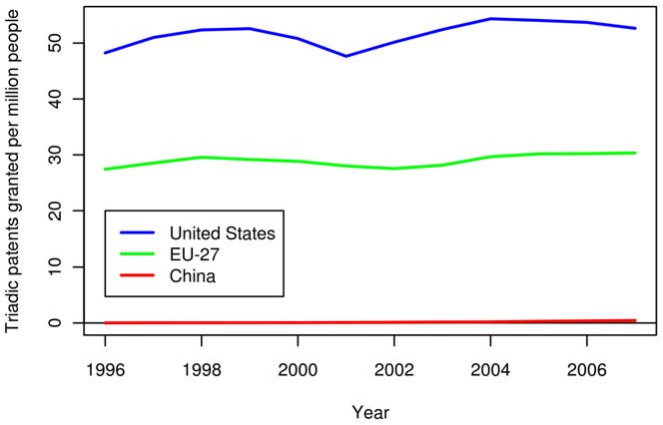
Per capita triadic patents granted in the US, the EU-27 and China. The blue, green, and red lines show the number of triadic patents granted per capita to American (US), European (within the EU-27), and Chinese inventors, respectively. Per capita figures were divided by the population of the region.

**Figure 13:**
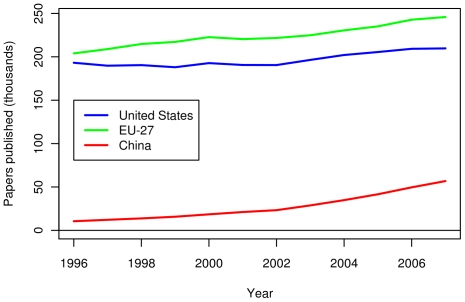
Recently published papers by researchers in the US, the EU-27 and China. Papers from journals included in the Science Citation Index and the Social Sciences Citation Index were enumerated. Each region received a fractional count based on the fraction of the institutions in the region.

**Figure 14:**
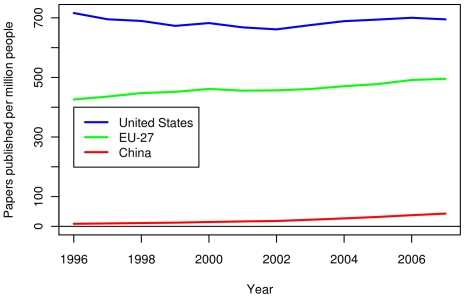
Published papers per capita by researchers in the US, the EU-27 and China. Papers from journals included in the Science Citation Index and the Social Sciences Citation Index were enumerated. Each region received a fractional count based on the fraction of the institutions in the region. The number of papers was divided by the size of the population of each region.

The triadic patent data in Figure 11 shows a close competition between the US and the EU in terms of absolute number of triadic patents. The number of triadic patents for China is far lower than both the US and EU. When normalized per capita, the US has a substantial lead over the EU in terms of triadic patents, and both still greatly lead China (see Figure 12). As triadic patents are not recognized and promoted in China, this metric is likely an uneven comparison, but analysis using domestic patent data showed similar trends. Figure 13 shows the number of papers published by authors in three compared regions between 1996 and 2007. The number of papers published per year is slowly increasing in the US. For the EU-27, the rate of increase is somewhat larger. The number of publications in China is the lowest among the three, but is increasing at the highest rate. Finally, while the EU-27 has more publications than the US in absolute number, the EU-27 has fewer publications per capita (see Figure 14).

## Discussion

Our findings can be summarized into eight key results. **1.** The level of US federal research funding (FRF) has varied between 0.2% and 0.6% of the GDP during the last six decades. **2.** Since the 1960s, the US FRF contribution has gone from twice that of industrial research funding to roughly equal. **3.** Similarly, since the 1960s, research and development (R&D) funding by the US government changed from almost twice that of industry to less than half of industry funding levels. **4.** The US FRF spending has also shifted in focus; approximately 65% of the total US R&D spending now goes to research support whereas in the 1960s ∼30% was directed to research support. **5.** There has been a decline in the fraction of spending devoted to research since 2001. **6.** Although well below the US and the EU in overall R&D funding, FRF in China has had a sustainable and high growth rate *vs.* stagnation in the US and the EU. **7.** The EU currently produces more science and engineering doctoral graduates and scientific publications than the US in absolute terms, but not per capita. **8.** One third of all the doctorate degrees in the US are obtained by the students on the temporary visas, and ∼40% of those return home within 5 years of their graduation [48].

Our world is an ever-changing environment, and it is naïve to think that any country can conduct business as it has been and expect that to be adequate for the future. While the US can pride itself on a legacy of remarkable advancements, it is time once again to reexamine what policies and resources are available for the future. We must examine the question: “What role is the US playing now, and what role will it play in the future of international science?” The US is facing increasing global competition in research and research related areas. Although our current comparisons are limited to China and the EU, it is clear that many regions throughout the world are investing in science and should be studied as well. These include other parts of Asia (specifically, India, Israel, Japan, South Korea, and South-West Asia), non-EU Europe (specifically, Russian Federation, Switzerland and Ukraine) and the Americas (specifically, Argentina, Brazil, Canada, and Mexico).

In addition, given the results of our analysis, we must consider “How will the US continue to foster its scientific strengths?” These results illustrate that the US financial commitment to research has plateaued in recent years, although the federal government has shifted more of its funding towards basic and applied research, while industry continues to concentrate on development. As science is founded on rigor and quality, it will be a mistake to be distracted by sheer quantity. A point in its favor is that the US currently has a very strong system of university research. In fact, in the 2009 Academic Ranking of World Universities, 17 of the top 20 universities were in the US [49]. However, at the same time, students in US K-to-12 schools are lagging behind students from many countries [50]. It is crucial that the US focus ever more diligently not only on the quality of the science it produces, but also on the quality of its scientific workforce. Therefore, focusing on K-12 education in general and, specifically, on STEM (Science-Technology-Engineering-Mathematics) becomes vitally important for the US [50].

Research (both basic and applied) translates into technological innovations that, in turn, transform into benefits for society and improvements in people's lives. Given that a substantial increase in funding is unlikely, the US government will have to find new innovative ways to increase the effectiveness of current funding. Similarly to post World War II, when Vannevar Bush helped to formulate new federal policy towards science [51], we argue that now is the best time to do the same. As the 21^st^ century moves ahead, it is vital that the federal government continues and strengthens its support of research and formulates a thoughtful and competitive science policy for this new century. This work calls for a serious discussion by the research communities within the government, academia and industry as well as among historians and administrators of science, policy analysts and makers (see, for example, [57]).
